# Evaluation of the Mechanism of Sinomenii Caulis in Treating Ulcerative Colitis based on Network Pharmacology and Molecular Docking

**DOI:** 10.2174/1573409919666230420083102

**Published:** 2024-09-26

**Authors:** Juan Tian, Changgeng Yang, Yun Wang, Canlin Zhou

**Affiliations:** 1 Life Science and Technology School, Lingnan Normal University, Zhanjiang, Guangdong, 524048, China

**Keywords:** Sinomenii Caulis, ulcerative colitis, network pharmacology, molecular docking, oxidative stress, inflammation

## Abstract

***Background*:** Studies have indicated that Sinomenii Caulis (SC) has several physiological activities, such as anti-inflammatory, anti-cancer, immunosuppression, and so on. SC is currently widely used in the treatment of rheumatoid arthritis, skin disease, and other diseases. However, the mechanism of SC in the treatment of ulcerative colitis (UC) remains unclear.

***Aims*:** To predict the active components of SC and determine the mechanism of SC on UC.

***Methods*:** Active components and targets of SC were screened and obtained by TCMSP, PharmMapper, and CTD databases. The target genes of UC were searched from GEO (GSE9452), and DisGeNET databases. Based on the String database, Cytoscape 3.7.2 software, and David 6.7 database, we analyzed the relationship between SC active components and UC potential targets or pathways. Finally, identification of SC targets in anti-UC by molecular docking. GROMACS software was used to perform molecular dynamics simulations of protein and compound complexes and to perform free energy calculations.

***Results*:** Six main active components, 61 potential anti-UC gene targets, and the top 5 targets with degree value are IL6, TNF, IL1β, CASP3, and SRC. According to GO enrichment analysis, the vascular endothelial growth factor receptor and vascular endothelial growth factor stimulus may be relevant biological processes implicated in the treatment of UC by SC. The KEGG pathway analysis result was mainly associated with the IL-17, AGE-RAGE, and TNF signaling pathways. Based on molecular docking results, beta-sitosterol, 16-epi-Isositsirikine, Sinomenine, and Stepholidine are strongly bound to the main targets. Molecular dynamics simulation results showed that IL1B/beta-sitosterol and TNF/16-epi-Isositsirikine binding was more stable.

***Conclusion*:** SC can play a therapeutic role in UC through multiple components, targets, and pathways. The specific mechanism of action needs to be further explored.

## 1 INTRODUCTION

Ulcerative colitis (UC) is an inflammation of the colon tissue and the intestinal submucosa Bowel disease. Clinically, the manifestations are abdominal pain, diarrhea, mucous pus, and blood stool, colonic ulcer surface slowly extends and causes the whole colonic ulcer in the end [1]. The pathogenesis of UC is affected by many factors, such as environmental, immune response disorder, epithelial barrier defect, and genetic susceptibility [2]. In the past, UC was mainly prevalent in Western countries mainly in Europe and North America [3]. However, since the end of the last century, the incidence of UC has risen rapidly in Asia and other developing countries with the lifestyle change [4, 5], it is worth noting that the annual incidence of UC in China is relatively high among Asian countries, with an average of 1.18/100,000 person-years [6]. UC is characterized by chronic recurrence and unpredictability, which not only affects the quality of life of patients but also causes a huge economic burden [7]. Therefore, the treatment of UC is particularly important. At present, the clinical treatment of UC mainly relies on Western medicine, but the therapeutic effect of Western medicine is not good and accompanied by gastrointestinal discomfort side effects [8].

Due to its advantages of low price, good curative effect, and few side effects, traditional Chinese medicine is used in the treatment of various diseases, including UC [9]. Sinomenii Caulis (qing-feng-teng in Chinese, SC), as a kind of traditional Chinese medicine, is the dried stems and rhizomes of *Sinomenium acutum* (Thunb.) Rehd. Pharmacological studies have indicated that SC has a wide range of physiological activities, such as anti-inflammatory, anti-cancer, immunosuppression, protection of organs from shock damage, and so on. Based on this, SC is currently widely used in the treatment of rheumatoid arthritis, skin disease, paralysis, and other diseases [10]. However, it is not clear whether SC can be used for the treatment of UC.

Network pharmacology is an emerging discipline that combines multiple disciplines and techniques to explore potential molecular mechanisms and relationships through the construction of biological network models [11]. Numerous studies have demonstrated the importance of network pharmacology in identifying drug targets and screening drug-active ingredients [12, 13]. In this study, we constructed a drug-target disease interaction network through network pharmacological analysis and verified the identified active components and key targets through molecular docking technology. The purpose of this study was to evaluate the therapeutic potential of SC on UC. The workflow is shown in Fig. (**1**).

## 2 MATERIALS AND METHODS

### 2.1 Screening SC and UC-related Targets

The reported chemical ingredient of SC is screen based on oral bioavailability (OB) ≥ 30% and drug similarity (DL) ≥ 0.18 through the TCMSP (https://tcmspw.com/tcmsp.php) database. Based on PharmMapper (http://www.lilab-ecus t.cn/pharmmapper/submitfile.htm) and the CTD database (http://ctdbase.org/), potential targets of UC can be predicted. The GEO database (https://www.ncbi.nlm.nih.gov/geo/), series GSE9452, was consulted for information regarding the differential DNA expression between normal subjects and UC patients. The microarray data was normalized with log2 (fold change) >2 or log2 (fold change) < -2 as the standard, which was considered to have significant differences in the expression. The UC-related genes were collected by integrating databases through searches of the DisGeNET database (https://www.disgenet.org/home/), followed by taking the intersection set between GEO and DisGeNET database. The potential targets of the UniProt database (https://www.uniprot.org ) were also used to standardize the names of all proteins and obtain the corresponding gene names.

### 2.2 Network Construction

To evaluate the interaction between SC and UC, we obtained a common target through Venny 2.1 (https://bioinfog p.cnb.csic.es/tools/venny/) and analyzed the targets using the STRING 11.0 (https://string-db.org/cgi/input.pl) database. The relationships between compounds of SC and targets of UC and protein-protein interactions were visualized using Cytoscape 3.7.2 software (https://cytoscape.org/).

### 2.3 GO and KEGG Pathway Enrichment Analysis

Data were uploaded to the DAVID database (http://david.abcc.ncifcrf.gov/) for analyses of GO functional enrichment and KEGG pathway enrichment. In this study, we screened relevant signal pathways on the premise of *p* < 0.05, and FDR < 0.05.

### 2.4 Molecular Docking

RCSB Protein Data Bank (http://www.rcsb.org/) was used to download the 3D protein structure of the core target, and Pymol software (http://www.pymol.org) was used to separate modified ligand, and dehydrated proteins. After downloading the 3D structure of the core component from PubChem (https://pubchem.ncbi.nlm.nih.gov/), adding hydrogen, and calculating the charge, Autodock Vina 1.1.2 software (https://autodock.scripps.edu/) was used to dock with the core target, and the limit binding energy ≤ -5.0 kcal·mol^-1^ was the stable connection site.

### 2.5 Binding Stability Verified by Molecular Dynamics Simulations

Simulations of molecular dynamics (MD) were carried out using GROMACS 2019.6 software. We optimized the simulation box size to have a distance greater than 1.0 nm between each atom of the protein and the box. Then, fill the box with water molecules based on a density of 1. To make the simulation system electrically neutral, the water molecules were replaced with Cl^-^ and Na^+^ ions. Using the steepest descent approach, 5.0104 stages of energy optimization were carried out to lower the overall system's energy consumption and, in the end, the unreasonably high contact or atom overlap. Following energy minimization, the system's temperature was stabilized via first-phase equilibration with the NVT ensemble at 300 K for 100 ps. The NPT ensemble was used to mimic second-phase equilibration at 1 bar and 100 ps. In an isothermal and isostatic ensemble with a temperature of 300 K and a pressure of 1 atmosphere, all MD simulations were run for 30 ns. The Parrinello-Rahman and V-rescale techniques, respectively, were used to manage the temperature and pressure, and the corresponding temperature and pressure coupling constants were 0.1 and 0.5 ps, respectively. The Van der Waals force was calculated using the Lennard-Jones function, and the nonbond truncation distance was set to 1.4 nm. The LINCS algorithm set restrictions on the length of each atom's connection. The long-range electrostatic interaction was calculated by the Particle Mesh-Ewald method with the Fourier spacing of 0.16 nm.

## 3 RESULTS

### 3.1 Common Targets Between SC and UC

**Fig. (2):**
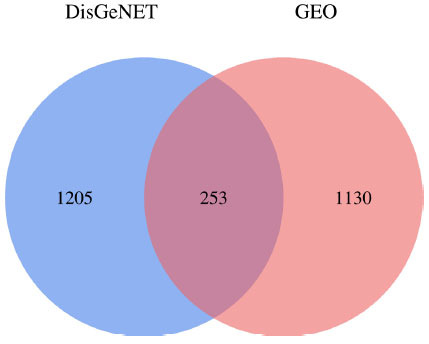
The union of disease targets in various databases.

**Fig. (3):**
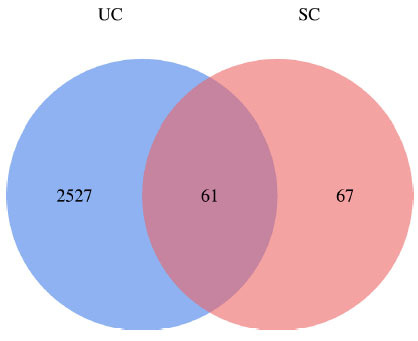
Component -disease target intersection diagram.

Sixteen chemical components of SC were retrieved from the TCMSP database (Table **S1**). After screening with the parameters OB ≥ 30% and DL ≥ 0.18, six active ingredients of SC, namely, beta-sitosterol, 16-epi-Isositsirikine, Magnograndiolide, Michelenolide, Sinomenine, and Stepholidine, were obtained for subsequent analysis (Table **1**). Among six active compounds, 128 targets were selected by the PharmMapper and CTD database after removing the repetitive targets. In addition, a total of 2588 UC-related targets were obtained by GEO and DisGeNET database (Fig. **2**). Then, the SC-related targets were mapped to UC-related targets by the Venn and we got 61 common targets (Fig. **3**, Table **2**).

### 3.2 PPI Network Construction and Hub Gene Screening

**Fig. (4):**
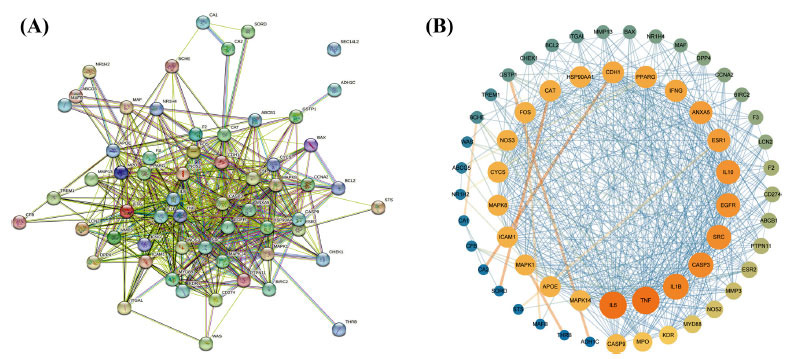
An association network of SC targeted proteins associated with UC. **(A)** The network for 61 co-targeted proteins had been selected as input for PPI analysis in STRING. **(B)** The PPI network for the core targets with the degree values range from large to small PPI networks.

To analyze hub targets in UC, we generated a network of co-targeted genes through the STRING database and visualized the network using Cytoscape 3.7.2, the results showed that the network included 59 nodes and 551 edges (Figs. **4A** and **B**). In general, genes with a higher degree are yellower, while genes with a lower degree are bluer. The top 5 hub genes were IL6, TNF, IL1β, CASP3, and SRC (Table **3**), indicating that these genes may be the target genes for the SC treatment of UC.

### 3.3 GO and KEGG Enrichment Analysis

From the DAVID Database, 186 GO items were obtained (*p* < 0.05, FDR < 0.05), of which 161 were BP, 9 CC, and 16 MF. In Fig. (**5A**), the top 10 memorably enriched GO terms were shown. Based on the results, SC targets displayed a strong connection with major biological processes, such as vascular endothelial growth factor receptor signaling pathway, cellular response to vascular endothelial growth factor stimulus, cellular response to reactive oxygen species, response to nutrient levels, negative regulation of the apoptotic process, innate immune response, positive regulation of transcription, DNA-templated. KEGG results show that a variety of signaling pathways may be its potential therapeutic mechanism. Sixty-one genes were enriched into a total of 114 signaling pathways, and the top 10 signaling pathways were shown in Fig. (**5B**). It was not difficult to find that IL-17, TNF, AGE-RAGE signaling pathway, Tuberculosis, Hepatitis B, and Toxoplasmosis were key pathways.

### 3.4 Molecular Docking Analysis

After removing the active ingredients with a degree value was less than 30 from Table **1**, the remaining four active ingredients were docked with the core targets protein. The docking results were shown in Table **4**, and the target protein with the best docking effect and corresponding compounds were shown in Fig. (**6**). There were no binding energies greater than -8.0 kcal/mol for the four compounds with TNF, indicating that they had a better docking effect.

### 3.5 RMSD, RMSF and Rg Results in Molecular Dynamics Simulations

The root mean square deviation (RMSD), which measures the deviation of the coordinates of a particular atom with respect to a reference structure, is often used to assess whether the simulation system has reached stability. A stable RMSD means that the corresponding atom becomes stable, while a fluctuating RMSD implies fluctuations. As shown in Fig. (**7**), all RMSDs have small fluctuating values during the simulation. This indicates stable binding of the protein ligands.

The RMSF calculates the rise and fall of each atom relative to its average position, characterizing the change in structure averaged over time, giving a characterization of the flexibility of each region of the protein. From Fig. (**8**), we can see that the CASP- beta-sitosterol complex group system has greater protein volatility than the other four groups.

The solvent accessible surface area (SASA) is calculated from the solute area by van der Waals forces interacting with solvent molecules. The solvent accessible surface area of a protein decreases with increasing protein tightness, so changes in SASA can predict changes in protein structure. As shown in Fig. (**9**), the SASA values for the protein-ligand complexes showed a decreasing trend during all the complex simulations. The decrease in solvent accessible and surface area values indicates that there is some shrinkage in both protein ligands, allowing fewer solvent molecules to access the surface region.

The radius of rotation (Rg) was used to demonstrate the tightness of the protein structure during the simulation, which is the distance between the centre of mass of all atoms and their ends for a given time interval. Fig. (**10**) shows that the Rg value of the protein decreases throughout the complex MD simulation, which is consistent with our SASA values, and therefore we conclude that the protein-ligand complex simulation process increases the tightness of the protein and the protein ligand is able to form stable complexes.

### 3.6 Combined Free Energy Calculation Results

Based on the trajectories of molecular dynamics simulations, we have calculated binding energies using the MMPBSA method, which can more accurately reflect the binding pattern of small molecules and target proteins. The results for the binding energy of the protein-ligand are shown in Table **5**, where the free energy of binding to the protein is -94.513 KJ/mol, -112.136 KJ/mol, -92.702 KJ/mol, -33.442 KJ/mol, and -124.665 KJ/mol. In the protein-ligand complex system, the binding energies are all negative, indicating that the molecule and the corresponding protein have a certain binding affinity.

## 4 DISCUSSION

UC is a chronic disease threatening human health, which has a certain correlation with colorectal cancer [14]. SC has anti-inflammatory, anti-cancer, immunosuppressive, and other physiological activities [15]. Sinomenine and beta-sitosterol, the main ingredients of SC, have been found to be effective in treating UC. However, whether SC can be used to treat UC remains unclear. Thus, further research is needed to discover unexplored therapeutic potential. Using network pharmacology in combination with multiple databases, this study examined whether SC is effective in treating UC. The study analyzed six core components with 128 SC targets and 2588 UC targets. There were 61 common targets, suggesting that the anti-UC targets of SC might be IL6, TNF, IL1B, CASP3, and SRC, and these targets were relatively high degrees in the PPI network. In addition, an enrichment analysis showed that core targets were mostly related to IL-17, AGE-RAGE, and TNF signaling pathways, *etc*. All of this suggests that SC may be therapeutic for UC.

As a result of this study, beta-sitosterol, 16-Epi-isositsirikine, Sinomenine, and Stepholidine have more targets than other components and are considered to be the core active components of SC. The effects of beta-sitosterol and Sinomenine on UC have been documented in many studies before [16, 17]. It has been reported that beta-sitosterol has immunomodulatory and anti-inflammatory properties. There has been extensive evidence that beta-sitosterol significantly reduces colon shortening, disease activity index (DAI), and the number of Th17 cells in the spleen in mouse experiments [18]. Sinomenine has been shown to prevent autoimmune and inflammation-related diseases *in vivo* studies [19, 20]. Moreover, sinomenine has also been shown to attenuate TNBS-induced colitis [21]. In previous studies, stepholidine has been shown to inhibit lipid peroxidation and scavenge hydroxyl radicals [22]. An imbalance between ROS and antioxidant defense in the colon contributes to the occurrence and progression of UC inflammation [23]. It is suggested that stepholidine may protect colon mucosa from injury by regulating the antioxidant/antioxidant balance. Studies on 16-Epi-isositsirikine are limited and there are reports suggesting that it has anticancer effects [24]. Nevertheless, the question of whether 16-Epi-isositsirikine plays a role in the development of UC still needs to be confirmed in the next step. Despite this, there is still much to learn about the specific mechanism of action. Therefore, the above active ingredients were considered important compounds of UC for SC treatment and have some reference value for subsequent research and application.

IL6, TNF, IL1β, CASP3, and SRC were regarded as major hub genes of SC on UC. IL1β is a pro-inflammatory factor, which is mainly secreted by monocytes, macrophages, and dendritic cells. It can trigger immune inflammation by releasing inflammatory chemokines. IL1β stimulates TNF production and *vice versa* [25]. TNF and IL1β are cytokines involved in early signal transduction and amplification induced by inflammatory response induction and activation of adhesion molecules, local inflammatory cell activation, and key enzymes of inflammatory response. The changes in intestinal mucosal function in UC patients were attributed to the increase of cytokines such as TNF and IL1β [26]. According to some scholars, IL1β also promotes the production of IL 6, which plays a role in IBD development by interacting with its receptors [27, 28]. IL-6 is a multi-effect cytokine, which can predict innate and adaptive immune responses. It is evaluated in patients' blood and colon mucosa and is positively correlated with UC activity [29]. It is reported that IL 6 can promote neutrophil chemotaxis, promote colon necrosis, and eventually destroy tissues [30]. SRC, an important tyrosine kinase, is predicted to be targeted by several kinds of tyrosine kinase inhibitors (TKIs). As a TKI targeting SRC, dasatinib shows powerful anti-inflammatory effects in UC rats induced with acetic acid [31]. Furthermore, SRC binds and activates PIK3R1 (p85), the driver of PI3K Signaling, resulting in activation of the NF- κB signaling cascade, which conduces to the sustained inflammatory response that damages intestinal tissue [32]. Molecular docking results indicated that the main active ingredients of SC, beta-sitosterol, 16-epi-Isositsirikine, Sinomenine, and Stepholidine, had a good affinity to the core target genes IL6, TNF, IL1β, CASP3, and SRC, suggesting that the key active components of SC have a therapeutic effect on UC. In addition, molecular dynamics simulations showed that IL1B/beta-sitosterol and TNF/16-epi-Isositsirikine binding was more stable.

An analysis of KEGG pathway enrichment revealed multiple signal pathways involved in the SC treatment of UC. This was evident in the IL-17, the Toxoplasmosis, the AGE-RAGE signaling pathway, Hepatitis B, and TNF signaling pathways, which were enriched in SC-associated hub gene targets closely related to UC. The IL-17 signaling pathway has been demonstrated to be one of the major factors contributing to UC occurrence and development. IL-17 is a specific effector from Th17 cells, which has a powerful inflammatory effect [33]. It facilitates the production of inflammatory factors such as IL-6 and IL-1β through the recruitment, mobilization, and activation of macrophages and neutrophils, mediating inflammatory invasion and tissue damage [34]. In addition to participating in oxidative stress signaling pathways, AGEs and RAGEs trigger ROS production, activate NF-κB, and cause inflammatory responses, and apoptosis [35]. IBD patients have RAGE polymorphisms and elevated RAGE levels, and their involvement in the inflammatory response is linked to NF-κB activation and its response to oxidative stress [36]. Among its many functions, the TNF signaling pathway regulates inflammation as well as the migration of intestinal epithelial cells [37] and demonstrates that TNF-α-induced receptor phosphorylation modulates intestinal wound healing in IBD by regulating ErbB signaling [38].

This is the first study to investigate the therapeutic potential of SC for UC. There are some limitations of our study that should be mentioned. First, other active components of SC may have been neglected due to limited database studies. Second, this study only focuses on the analysis of existing databases and lacks verification experiments at the cellular or molecular level.

## CONCLUSION

All in all, SC can play a therapeutic role in UC through multiple components, targets, and pathways. At the same time, it was found that the key active components of SC mainly function by interfering with key signaling targets like inflammation and oxidative stress in the treatment of UC. Nevertheless, the specific mechanism of action needs to be further explored.

## Figures and Tables

**Fig. (1):**
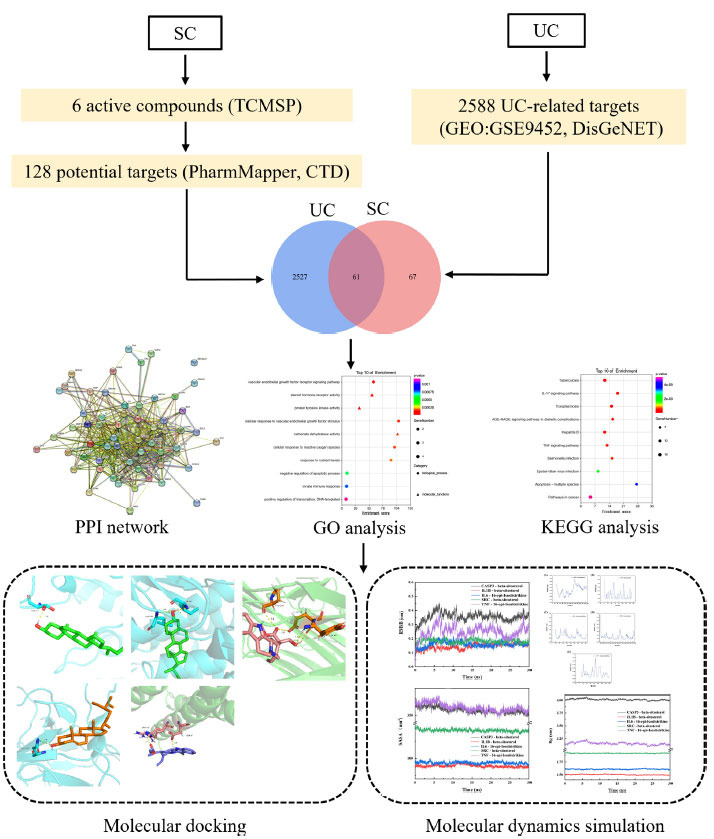
The detailed flow chart of the current study.

**Fig. (5):**
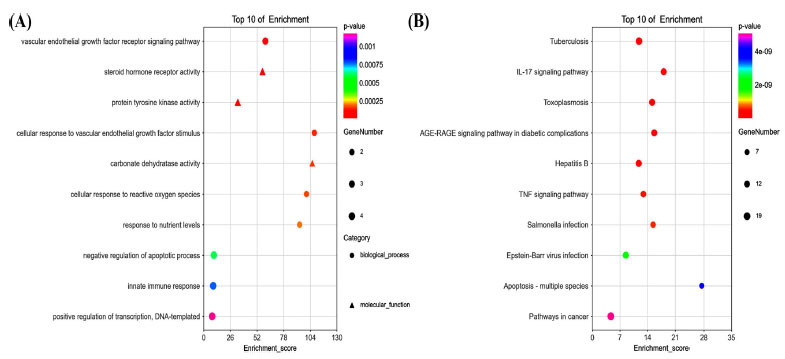
Gene Ontology (GO) enrichment and KEGG pathways analysis for 61 drug-disease targets. **(A)** The top 10 significant GO enrichment analyses. **(B)** The top 10 significant KEGG pathways.

**Fig. (6):**
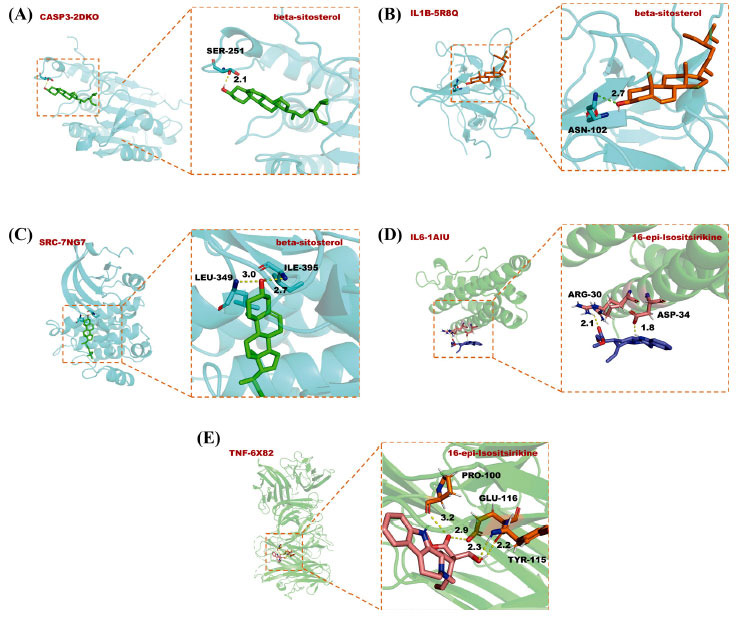
Analysis of the binding mode of the best docking conformation. (**A**) CASP3 and beta-sitosterol; (**B**) IL1β and beta-sitosterol; (**C**) SRC and beta-sitosterol; (**D**) IL6 and 16-epi-Isositsirikine; (**E**) TNF and 16-epi-Isositsirikine.

**Fig. (7):**
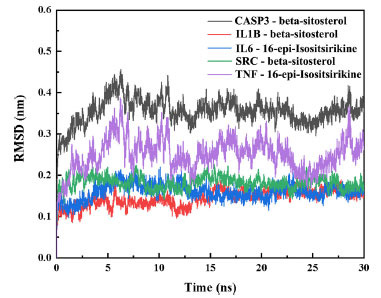
Curve of RMSD of protein with time during protein-ligand complex simulation.

**Fig. (8):**
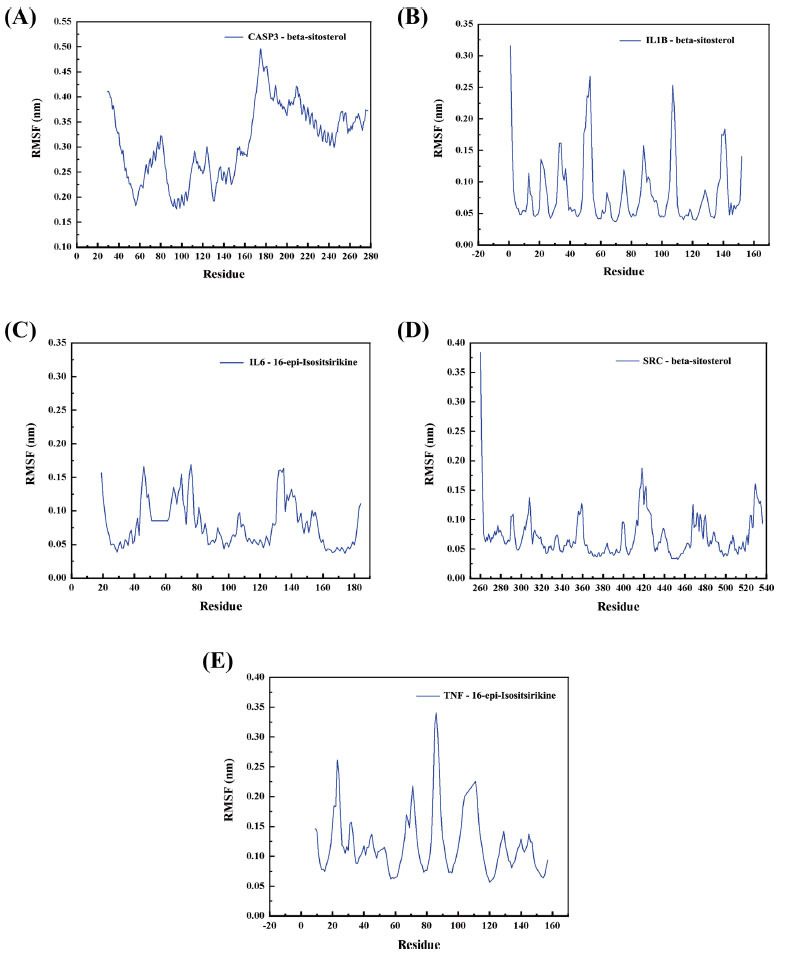
Protein RMSF profiles with time during protein-ligand complex simulation. (**A**) CASP3 and beta-sitosterol; (**B**) IL1β and beta-sitosterol; (**C**) IL6 and 16-epi-Isositsirikine; (**D**) SRC and beta-sitosterol; (**E**) TNF and 16-epi-Isositsirikine.

**Fig. (9):**
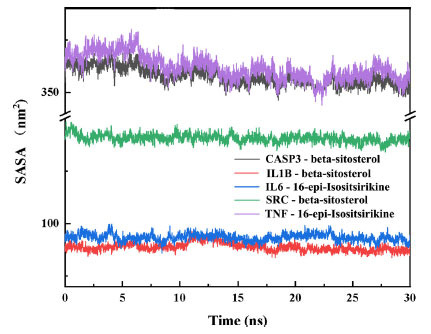
SASA profiles of proteins with time during protein-ligand complex simulation.

**Fig. (10):**
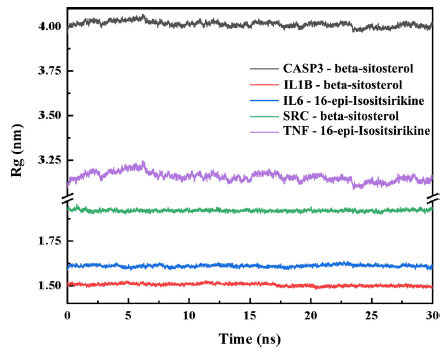
Rg profiles of proteins with time during protein-ligand complex simulation.

**Table 1:** Active compounds of SC screened by TCMSP.

**Mol ID**	**Molecule Name**	**PubChem ID**	**OB (%)**	**DL**	**Degree**	**Structure**
MOL000358	Beta-sitosterol	12303645	36.91	0.75	71	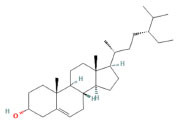
MOL000621	16-epi-Isositsirikine	6436828	49.52	0.59	50	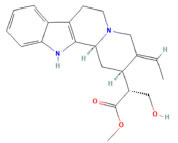
MOL000622	Magnograndiolide	5319198	63.71	0.19	4	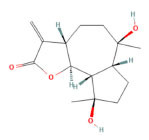
MOL000623	Michelenolide	442278	47.54	0.25	15	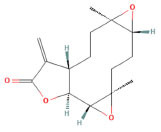
MOL000625	Sinomenine	5459308	46.09	0.53	32	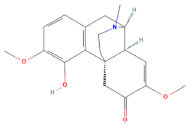
MOL000627	Stepholidine	12442999	33.11	0.54	36	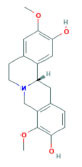

**Table 2:** Information about 61 common targets.

**ABCG5**	**CDH1**	**ANXA5**	**MAPK14**	**SRC**	**CFB**	**IFNG**	**ABCB1**
APOE	CYCS	CA2	MAPK8	STS	HSP90AA1	MAF	MYD88
BAX	ESR1	CHEK1	MMP13	THRB	MMP3	TNF	MAFB
BCL2	ESR2	EGFR	NOS3	WAS	TREM1	MIR155	FOS
BIRC2	ICAM1	F2	NR1H4	ADH1C	DPP4	MPO	F3
CASP3	IL10	ITGAL	PPARG	BCHE	GSTP1	NOS2	LCN2
CASP9	IL1B	KDR	PTPN11	CA1	NR1H2	CD274	SORD
CAT	IL6	MAPK1	SEC14L2	CCNA2	-	-	-

**Table 3:** Information on 5 core targets.

**Name**	**DC**	**BC**	**CC**
TNF	44	0.065622452	0.794520548
IL6	44	0.065622452	0.794520548
IL1β	40	0.034692882	0.743589744
CASP3	38	0.031016357	0.725
SRC	37	0.032760898	0.716049383

**Table 4:** Docking scores of the active ingredients with their potential targets.

**-**	**TNF**	**IL6**	**IL1β**	**CASP3**	**SRC**
Beta-sitosterol	-8.09	-7.47	-7.9	-8.25	-8.48
16-epi-Isositsirikine	-8.95	-7.68	-7.6	-7.41	-7.91
Sinomenine	-8.31	-6.82	-7.29	-7.15	-7.22
Stepholidine	-8.26	-6.76	-7.06	-7.36	-7.8

**Table 5:** Analysis of the protein-ligand MMPBSA (KJ/mol).

**Energy**	**CASP3/beta-sitosterol**	**IL1B/beta-sitosterol**	**IL6/16-epi-Isositsirikine**	**SRC/beta-sitosterol**	**TNF/16-epi-Isositsirikine**
Van der Waals energy	-146.748	-165.419	-131.003	-173.121	-165.136
Electrostatic energy	-91.387	-61.786	6.784	-15.126	-15.389
Polar solvation energy	161.739	132.060	45.715	175.315	75.335
Nonpolar solvation energy	-18.118	-16.991	-14.784	-20.511	-19.474
Total Binding energy	-94.513	-112.136	-92.702	-33.442	-124.665

## Data Availability

All data generated or analyzed during this study are included in this manuscript.

## LIST OF ABBREVIATIONS

DAI: Disease Activity Index
SASA: Solvent Accessible Surface Area
TKIs: Tyrosine Kinase Inhibitors
UC: Ulcerative Colitis
