# Supplementary material for: Evaluation of the Mechanism of Sinomenii Caulis in Treating Ulcerative Colitis based on Network Pharmacology and Molecular Docking
Source: Curr Comput Aided Drug Des. 2024 Sep 26;20(3):195–207. doi: 10.2174/1573409919666230420083102 (PMC10641851; doi:10.2174/1573409919666230420083102)
Supplement: Supplementary file 1 — Supplementary material is available on the publisher’s website along with the published article. [file CCADD-20-195_SD1.pdf]

Supplementary Material

Evaluation of the Mechanism of Sinomenii Caulis in Treating Ulcerative Colitis based on Network Pharmacology and Molecular Docking

Juan Tian[1], Changgeng Yang[1], Yun Wang[1] and Canlin Zhou[1,*]

[1]*Life Science and Technology School, Lingnan Normal University, Zhanjiang, Guangdong, 524048, China*

Table S1. Sixteen chemical components of SC were retrieved from the TCMSP database.

Mol ID	Molecule Name	OB (%)	DL
MOL000358	Beta-sitosterol	36.91	0.75
MOL000365	Syringaresinol	3.29	0.72
MOL000616	(+)-Suyringaresinol-di-O-beta-D-glucoside	5.19	0.29
MOL000617	(14S)-14-methylpalmitic acid	23.12	0.11
MOL012907	Acutumidine	2.04	0.41
MOL000619	Acutumine	2.36	0.44
MOL000620	Dispegatrine	8.66	0.03
MOL000621	16-epi-Isositsirikine	49.52	0.59
MOL000622	Magnograndiolid	63.71	0.19
MOL000623	Michelenolide	47.54	0.25
MOL000624	[[2-(4-hydroxyphenyl)-1-[(2S,3R,4S,5S,6R)-3,4,5-trihydroxy-6-(hydroxymethyl)oxan-2-yl]sulfanylethylidene]amino] hydrogen sulfate	3.97	0.35
MOL000625	Sinomenine	46.09	0.53
MOL000626	Sinomontanine D	14.05	0.66
MOL000627	Stepholidine	33.11	0.54
MOL000628	Darutoside	21.32	0.63
MOL000629	Tufulingoside	24.06	0.35
